# Comparison of Different Obturation Techniques in the Dentinal Tubule Penetration of EndoSequence® Bioceramic Sealer HiFlow™: An In-Vitro Study

**DOI:** 10.7759/cureus.71155

**Published:** 2024-10-09

**Authors:** Mohammad Mobayed, Helen R Ayoubi, Hassan Achour, Yasser Alsayed Tolibah

**Affiliations:** 1 Department of Endodontics, Damascus University, Damascus, SYR; 2 Department of Endodontics and Operative Dentistry, Faculty of Dental Medicine, Damascus University, Damascus, SYR; 3 Department of Pediatric Dentistry, Damascus University, Damascus, SYR

**Keywords:** bioceramic sealer hiflow, dentinal tubule penetration, maximum penetration depth, obturation technique, scanning electron microscope

## Abstract

Background

This study aimed to assess the dentinal tubule penetration of bioceramic sealer (DTPB) using EndoSequence® Bioceramic Sealer HiFlow™ (BCHF). Three obturation techniques were compared, namely, single cone (SC), cold lateral condensation (CLC), and warm vertical compaction (WVC) using scanning electron microscopy (SEM).

Methodology

A total of 45 intact mandibular premolars were decoronated, instrumented, and irrigated with uniform procedures. Subsequently, they were randomly assigned into the following three groups according to the gutta-percha and BCHF obturation technique: Group 1 (n = 15) with the SC technique, Group 2 (n = 15) with the CLC technique, and Group 3 (n = 15) with the WVC technique. Afterward, the roots were sectioned at three levels (coronal, middle, apical) from the apex and examined using SEM to determine the maximum distance of DTPB in µm. The collected data were analyzed using SPSS software (IBM Corp., Armonk, NY, USA) using the one-way analysis of variance test and Bonferroni tests. The level of significance was set at α of 0.05.

Results

Significant differences were observed in the maximum distances of DTPB values among the three levels across groups (p < 0.05). Both the WVC and CLC groups exhibited higher values compared to the SC group (p < 0.05). Specifically, the DTPB maximum distance at the apical third was significantly higher in the CLC group than in the WVC and SC groups (p < 0.05). Additionally, the coronal third in each group demonstrated significant DTPB maximum distance (p < 0.05) compared to the other groups.

Conclusions

BCHF with the WVC and CLC obturation technique showed the best performance in DTPB maximum distance values at the three levels.

## Introduction

Three-dimensional (3D) obturation of the root canal system provides a tight seal to prevent leakage that can lead to subsequent failure of endodontic treatment [1]. Traditionally, gutta-percha (GP) is used in combination with an endodontic sealer (ES) to ensure the adaptation and complete sealing of the root canal system. The ES should create a sufficient seal with the GP and dentine surface to reduce gaps [2].

Microscopically, the root dentine consists of a large number of dentinal tubules. It is worth mentioning that the dentinal tubules in human premolars are denser in the coronal and apical third of the canal compared to the middle third [3]. Therefore, the sealing ability of the ESs into the dentinal surface may reflect how they can penetrate dentinal tubules and form a physical barrier by chemical bonding and mechanical locking [4]. This procedure is affected by the root canal morphology, irrigation protocol, physiochemical properties of the ES, ES activation, and obturation method used [5].

The resin-based sealer has been used as a golden standard ES due to its apical sealing ability, low solubility, and easy handling. However, since the 1990s, there has been an increasing focus on bio-materials such as calcium silicate-based materials in endodontics. These materials have emerged as an area of research and clinical applications in dentistry due to their biocompatibility, superiority, and comparable sealing ability compared to resin-based sealers [6].

EndoSequence® BioCeramic Sealer™ (BS) (Brasseler USA, Savannah, GA, USA) was introduced in 2009. It is a premixed injectable ES that consists of zirconium oxide, calcium silicates, calcium hydroxide, calcium phosphate, filler, and thickening agents [7]. This product has been shown to have low cytotoxicity, adequate bonding strength, and favorable sealing ability [8]. BS can be used with the single cone (SC), cold lateral condensation (CLC), or warm vertical compaction (WVC) obturation techniques [9]. On the other hand, Yamauchi et al. indicated that heating calcium silicate cement such as EndoSequence® BS in terms of the WVC technique could affect the physical properties of this sealer, where heat can reduce the setting time, decrease the flow, and increase the film thickness of EndoSequence® BS [10]. Therefore, a novel premixed BC (EndoSequence® BC Sealer HiFlow™ (BCHF), Brasseler USA, Savannah, GA, USA) has been popularized to fit with the WVC technique and overcome the heat drawbacks, where it showed better performance in comparison with BS when using the WVC as a root canal obturation technique [11]. To our knowledge, there were few published data on the dentinal tubule penetration of BCHF (DTPB) using SC and WVC obturation techniques assessed under a confocal laser microscope (CLSM) [12-15], and one that assessed the overall dentinal penetration under scanning electron microscope (SEM) [15]. Therefore, this study aimed to assess the maximum distance of DTPB at three distinct levels of the root canal (apical, middle, and coronal third) when employing BCHF with SC, CLC, or WVC obturation techniques under SEM examination. The null hypothesis posits that there is no significant variance in DTPB following the utilization of the three specified obturation techniques across the three levels.

## Materials and methods

Study design and ethical considerations

This experimental in-vitro study was conducted following the ethical guidelines of the Declaration of Helsinki. The research project was approved by the Local Research Ethics Committee, Faculty of Dentistry, Damascus University (approval number: UDDS-693-60122021/SRC-1550), and was funded by Damascus University (funder number: 501100020595). Patients provided informed consent that their extracted teeth would be examined in this study.

Sample size calculation

Drawing from the findings of a previous investigation [14], the sample size for this study was determined utilizing G* Power 3.1.9.4 (Heinrich-Heine-Universität, Düsseldorf, Germany). In the analysis of variance (ANOVA) analysis, sample sizes of 15 were derived for each of the three groups, resulting in a total sample of 45 subjects. This configuration yielded an effect size (f) of 0.4811 (which was calculated according to the change in the maximum penetration depth in mm), the maximum, and 80% power to discern disparities at a significance level of 0.05.

Inclusion and exclusion criteria

This study involved 45 freshly extracted human, single-rooted, intact, mature, permanent, mandibular first premolar that were extracted for orthodontic reasons. After extraction, the premolars were examined with a 2.5 magnification lens (Carson handheld, Ronkonkoma, NY, USA) to ensure that they were free of cracks. Any visible calculus was removed using ultrasonic tips (Eighteeth, Changzhou, China). Two periapical radiographs (buccolingual and mesiodistal) were taken for each premolar to ensure that it was free of anatomical abnormalities with type I of Vertucci classification. Finally, the teeth were kept for two hours in 4% NaOCl (Shahabamed, Aleppo, Syria), and then stored in normal saline. Premolars with root resorption, root curvature, immature apex, fracture, or previous obturation were excluded.

Sample preparation

The selected premolars were decoronated at 15 mm from the apex by a double-faced diamond disk (Hager & Meisinger, Neuss, Germany) mounted on a low-speed straight handpiece to standardize sample lengths and facilitate canal instrumentation. The canal orifice was prepared using an orifice opener (SX) file (ProTaper Universal Rotary File; Dentsply, Maillefer, Ballaigues, Switzerland), and then the patency was checked with a 10 K-file (Mani, Utsunomiya, Japan) until the tip was visible at the apices. The working length (WL) was established by subtracting 0.5 mm from this measurement. Afterward, the root canals were prepared up to F3 file (ProTaper Rotary File; Dentsply, Maillefer, Ballaigues, Switzerland) mounted on Endodontic Rotary Motor (X-Smart; Dentsply Sirona, Charlotte, NC, USA) according to the manufacturer’s instructions (400 rpm, 4 Ncm). During instrumentation, the root canals were irrigated with 10 mL of 5.25% NaOCl. After instrumentation, the canals were irrigated with 10 mL of 17% ethylenediaminetetraacetic acid (EDTA), followed by 3 mL of 5.25% NaOCl with ultrasonic activation for one minute using an ultrasonic tip (Woodpecker, Shanghai, China), and a final flush with 10 mL of normal saline. Irrigating solutions were delivered using a 27-gauge side-vented needle (Ultradent Products, Inc., South Jordan, UT, USA). The root canals were then dried with paper points (DiaDent, Chongju, Korea). Finally, each root was covered with soft modeling wax (Cera Reus SA, Reus, Spain) and embedded in an acrylic block (5 × 5 × 15 mm) to simulate the periodontium during canal obturation and facilitate sample segmentation at three levels to be subjected to SEM examination. During this procedure, a standard 30.06 master GP cone (DiaDent, Chongju, Korea) was introduced into the canal to the WL to prevent wax penetration into the canal space, following which it was removed.

Sample distribution and root canal obturation

Each premolar received a unique identifier number randomly assigned to the three study groups using the randomization.org website.

Each canal was dried with (30.06) paper points and filled individually. Afterward, samples were randomly divided into the following three groups according to the obturation technique: Group 1 (n = 15) was filled with the SC technique, Group 2 (n = 15) was filled with the CLC technique, and Group 3 (n = 15) was filled with the WVC technique. Within all groups, a standard 30.06 master GP cone (DiaDent, Chongju, Korea) was chosen for each premolar, with each demonstrating tug-back at the WL. Moreover, BCHF was injected into the root canal up to 4 mm short of the WL using plastic syringes and needles provided by the manufacturer in all groups.

Single Cone Technique

After injecting BCHF, the master cone was slowly inserted into the canal to its WL. Then, the excess GP was trimmed off with an electrical heat carrier 1 mm below the orifice and vertically compacted with a hand plugger.

Cold Lateral Condensation Technique

A spreader (25.02) was pre-fitted to ensure that it could be inserted into 1-2 mm before the WL. After injecting BCHF, a standard CLC technique was performed, where the spreader was placed into its maximum depth and then removed by rotating it back and forth as it was withdrawn. The accessory cones (25.02 and 20.02) were placed in the space vacated by the spreader. The process was repeated until the spreader could be inserted no more than 2 mm (from the orifice of the canal).

Warm Vertical Compaction Technique

The WVC technique was used, where the fastback tip was pre-fitted to reach 4-5 mm before the WL, and the device temperature (Fast Fill; Eighteeth, Changzhou, China) was set at 200°C. After injecting BCHF, 0.5 mm of the master cone was clipped to avoid exceeding the thermoplastic GP outside the apex. Then, it was inserted into the canal, the fastback tip was inserted with a one-way insertion motion, and taken to a depth of 4-5 mm before the WL. The tip was allowed to cool for 15 seconds, following which a single burst of heat was applied for one second, and then the tip was removed. This procedure was repeated twice: the first to the middle third of the canal, and the second to the coronal third of the canal. Finally, the excess GP was removed from the canal orifice and then compacted with a 0.6 mm (No ½) hand plugger (Dentsply, Tulsa, OK, USA).

Afterward, the canal orifice was restored using a temporary filling (Coltozol; Coltene, Raiffeisentrabe, Germany). The samples were kept in a moist environment (100% humidity) in a special incubator (Binder, Tuttlingen, Germany) at 37°C for 14 days. Subsequently, samples were cross-sectioned with a low-speed fan-empty diamond disc with a thickness of 0.25 mm at a slow rotational speed of 25,000 cycles per minute under heavy water cooling at three levels, namely, coronal, middle, and apical (5 mm for each level). A thickness of 1.5 mm was adopted for each section, and the samples were placed in a water bath for one minute at a 45°C temperature. This ensured minimal generation of the smear layer without causing damage to the obturation material.

Scanning electron microscope analysis

The samples obtained were dehydrated using the following regime: 70% alcohol for 12 hours, 80% alcohol for 12 hours, 90% alcohol for six hours, and 99.7% alcohol for three hours. Afterward, they were soaked in a 5% nitric acid solution for three minutes to obtain a clean surface residual debris (such as a smear layer), following which they were rinsed with distilled water and left for 24 hours to dry [16].

**Figure 1:**
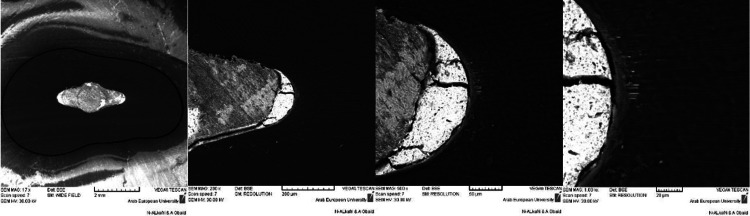
The investigation of DTPB observed in the same sections (the lower magnification at the left to the higher magnification at the right). DTPB: dentinal tubule penetration of bioceramic sealer

**Figure 2:**
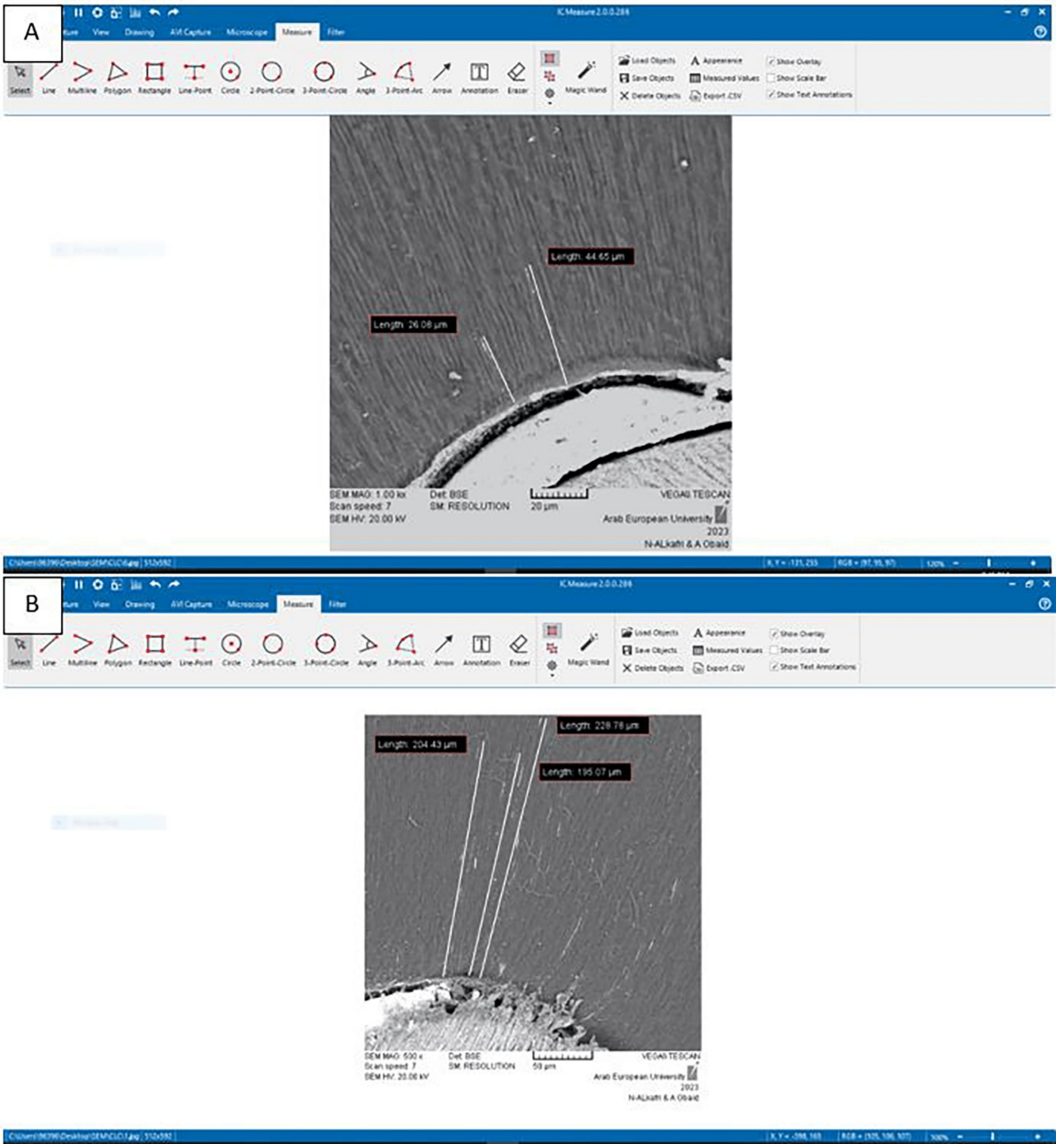
Multi-measures of dentinal penetration depth (um) using the IC Measure software of some samples under SEM to determine the DTPB maximum distance. A: apical third; B: middle third. DTPB: dentinal tubule penetration of bioceramic sealer; SEM: scanning electron microscopy

Each section underwent an SEM (90-10,000× magnification) (VEGAII-XMU, Tescan, Czech Republic) examination under different magnifications (Figure 1), capturing digital images of areas where sealer penetration in the dentinal tubules was observed. This procedure was repeated within all sections for the three levels in each group. Subsequently, the distance from the root canal surface to the deepest extent of the visible sealer was calculated in µm using IC software (IC Measure, The Imaging Source, Charlotte, NC, USA). The maximum distance in each section was considered the maximum DTPB (Figure 2). Measurements were performed by two blinded operators (2 PhD Students in the Department of Endodontics) who were pre-trained in SEM analysis. This measurement method is similar to the one described by Schmidt et al. [17] and Marissa et al. [18]. However, the sections were not divided into quadrants, as was done by Schmidt et al., to avoid any further distortion of the sections.

Statistical analysis

The collected data were tabulated and analyzed using SPSS software (Version 13, SPSS Inc., Chicago, IL, USA). Kolmogorov-Smirnov and Shapiro-Wilk tests indicated the normal distribution of DTPB in µm among the three groups at each third (p > 0.05). Moreover, homogeneity of variance was assessed using the Levene test which indicated that variances were homogeneous among groups (p > 0.05); hence, intergroup and intragroup comparisons regarding the DTPB in µm at each third were performed using the one-way analysis of variance (ANOVA test, and the pairwise comparisons were performed using the Bonferroni test. The level of significance was set at α of 0.05.

## Results

The sample consisted of 45 mandibular premolars, selected, prepared, and irrigated under as uniform conditions as possible and divided equally into three groups based on the obturation technique used. The samples were obturated using GP cones and BCHF with different techniques, namely, SC, CLC, and WVC. No loss of samples occurred throughout the study. The quantitative results of the SEM analysis in terms of DTPB maximum distance in µm at the three levels among the three groups and the one-way ANOVA test are presented in Table 1.

**Table 1:** Mean, standard deviation, and range of maximum distance of dentinal tubules penetration of BCHF in µm at each level (coronal, middle, and apical) among groups and the one-way ANOVA test results. Groups with the same small letters in the same rows indicate significant pairwise differences. Groups with the same Latin numbers in the same column indicate significant pairwise differences. *: one-way ANOVA test; SC: single cone; CLC: cold lateral condensation; WVC: warm vertical compaction; ^: significant differences. BCHF: Bioceramic Sealer HiFlow™; SD: standard deviation; ANOVA: analysis of variance

Level/Group	SC group		CLC group		WVC group		Comparisons between groups at each level (*p-value)
	Mean ± SD	Range	Mean ± SD	Range	Mean ± SD	Range	
Coronal third	75.70 ± 39.14 µm^a, I^	27.4–171.3 µm	135.63 ± 51.10 µm^b, I, II^	72.3–249.9 µm	205.16 ± 136.02 µm^a, b, I, II^	79.9–529.8 µm	<0.001^
Middle third	64.52 ± 20.04 µm^a, II^	24.1–97.1 µm	70.36 ± 26.98 µm^b, I^	30.8–113.5 µm	97.03 ± 25.39 µm^a, b, I^	59.3–138.3 µm	<0.001^
Apical third	41.63 ± 18.24 µm^a, I, II^	13.2–171.3 µm	71.98 ± 22.73 µm^a, b, II^	30.1–116.3 µm	52.75 ± 24.37 µm^b, II^	18.2–107.1 µm	<0.001^
Comparisons between thirds at each group (*p-value)	0.001^		<0.001^		<0.001^		

The one-way ANOVA test showed significant differences in DTPB maximum distance in µm between the groups at each level (p < 0.001 at each third).

The Bonferroni test showed that, at the coronal third, the WVC group had the highest mean of DTPB maximum distance (205.16 µm) compared with that of the CLC and SC groups, and the differences were statistically significant (135.63 µm and  75.70 µm; p = 0.042 and p < 0.001, respectively). Similarly, at the middle third, the WVC group had the highest mean of DTPB maximum distance (97.03 µm) compared with that of the CLC and SC groups, and the differences were statistically significant (70.36 µm and 64.52 µm; p = 0.003 and p < 0.001, respectively). However, at the apical third, the CLC group had the highest mean of DTPB maximum distance (71.98 µm) compared with that of the WVC and SC groups, and the differences were statistically significant (52.75 µm and 41.63 µm; p = 0.023 and p < 0.001, respectively).

The one-way ANOVA test showed significant differences in DTPB maximum distance in µm between the thirds in each group, whereas the Bonferroni test showed that the WVC and CLC groups showed better DTPB in the coronal third with significant differences in comparison with middle and apical thirds (p < 0.001 in each pairwise comparison). However, in the SC group, the apical third showed the lowest DTPB maximum distance in comparison with the coronal and middle thirds (p = 0.001, and p = 0.033, respectively).

**Figure 3:**
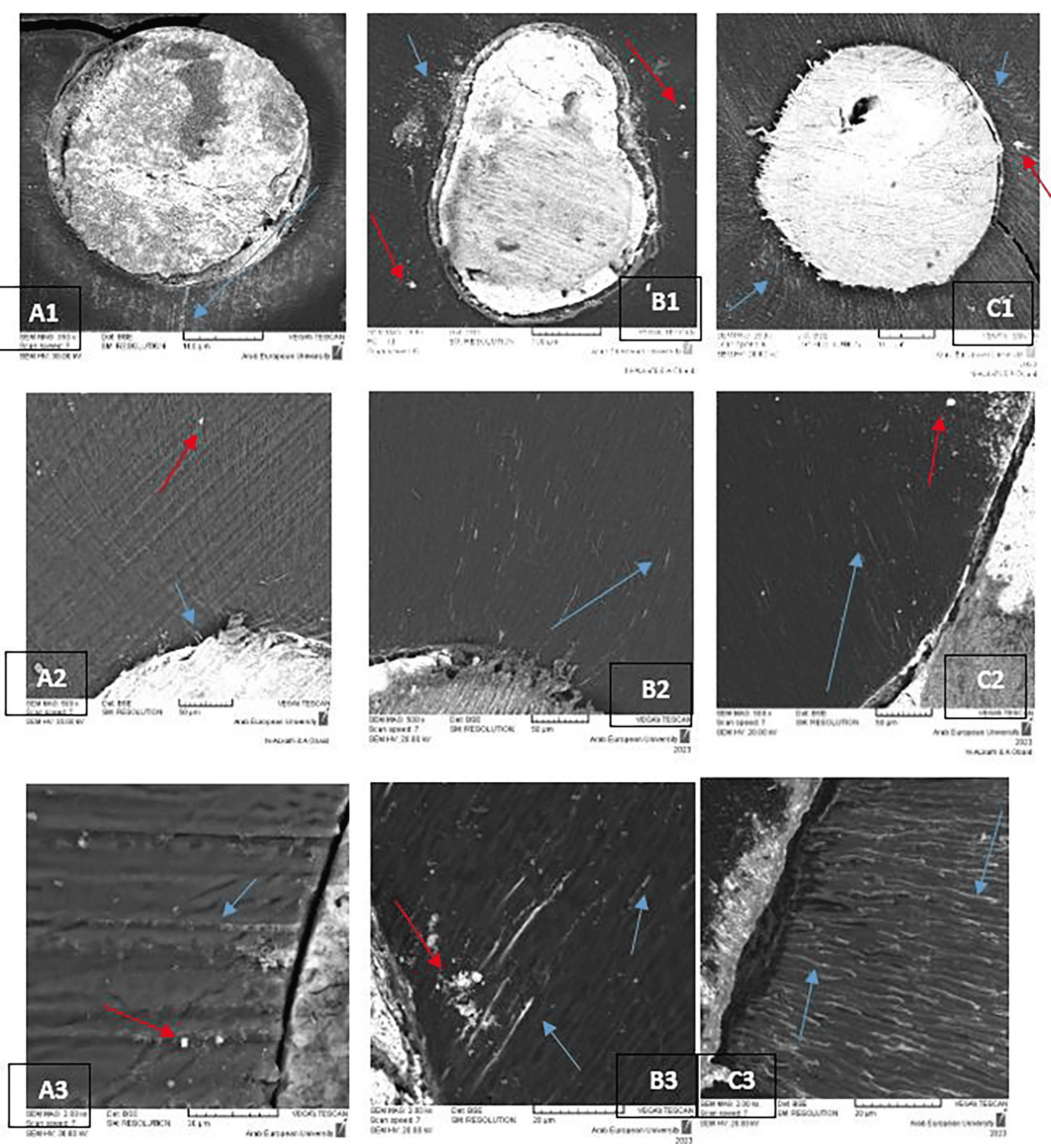
Representative SEM of sealer’s depth penetration in the dentinal tubules. (A) SC group (A1 ×350 for the coronal third; A2 ×500 for the middle third; A3 ×2,000 for the apical third). (B) CLC group (B1 ×300 for the coronal third; B2 ×500 for the middle third; B3 ×2,000 for the apical third). (C) WVC group (C1 ×250 for the coronal third; C2 ×500 for the middle third; C3 ×2,000 for the apical third). Blue arrows indicate detected DTPB, while red arrows indicate detected sealer’s artifact. DTPB: dentinal tubule penetration of bioceramic sealer; SEM: scanning electron microscopy; SC: single cone; CLC: cold lateral condensation; WVC: warm vertical compaction

Figure 3 shows some SEM images of DTPB maximum distance at the three levels among the three groups.

## Discussion

A recent systematic review that discussed the calcium silicate-based sealer penetration into dentinal tubules noted dissimilarities among the studies assessing the obturation techniques and suggested further investigation in this regard [5]. The novelty of this study resides in exploring the effect of three obturation techniques (SC, CLC, and WVC) using GP and BCHF on the dentinal tubule penetration at three levels of the root canal under SEM examination to detect the best hermetic seal of the root canal system for better long-term outcomes.

Although single-rooted premolars with single canals were selected for this study due to their simplified canal anatomy, the aim was to standardize the criteria to provide an accurate depiction of the effect of the canal obturation technique on the BCHF. This could later be extrapolated to more complex anatomized teeth.

A specific methodology was followed in this study. During the selection process for inclusion and exclusion criteria, premolars were carefully chosen to ensure uniformity in shape radiographically with similar lengths, aiming to standardize root canal preparation and obturation procedures. NaOCl was used because it is available and commonly used. The 5.25% concentration was adopted because it is the most widely used in endodontic treatments and can dissolve normal and necrotic pulp tissue and the biofilm layer inside the root canal system [18]. Moreover, 17% EDTA solution was used as a chelating agent to remove the smear layer due to its ability to remove calcium ions from the dentin and thus dissolve the inorganic components from the smear layer [19]. It is worth noting that the chelating action of EDTA helped to obtain the highest penetration of BS into dentinal tubules [20]. Additionally, ultrasonic activation has a high effectiveness in removing the smear layer and opening the dentinal canals [21], which may increase the DTPB. After instrumentation procedures, a layer of grade wax was placed on the external root walls to mimic the presence of the periodontal ligament in natural teeth, where the insertion of the obturation material into the canals will be affected by the absence of this layer due to the difference in pressure inside the canal.

Sealer penetration into dentinal tubules is usually detected using the CLSM, where sealers are mixed with organic dyes such as rhodamine B and indirectly detected by fluorescence of the organic dye [12,13,15]. Research has shown that the depth of rhodamine B dye penetration does not align with the detection of sealer within the dentinal tubules under SEM analysis [22]. Specimen preparation for SEM analysis, including sawing, might impact the outcomes, potentially causing the extrusion of ES from the tubules. In this study, DTPB was primarily near the main root canal, and there was no evidence of sealer loss from the tubules. However, it is important to note that this method is restricted to the specimen’s surface and provides visibility into only a fraction of all dentinal tubules within the root dentin [17,23].

Fortunately, while preparing cross-sectional samples from the included premolars and subsequently examining them under SEM, no cross-sections exhibiting the butterfly effect were observed. This effect serves as a potential confounder during the assessment of sealer penetration, as sections displaying this effect typically indicate greater penetration in the buccolingual direction than in the mesiodistal direction [24].

Although some cross-sectional images (such as A3, B3, and C2 panels in Figure 3) exhibited the sealer’s artifact, which may have resulted from the cutting procedures, it was still possible to distinguish them from sealer penetration based on the continuity of shape and direction.

The maximum penetration distance was adopted as a measurement method to analyze the SEM cross-sections, as it has been used in several previous studies [25,26]. This choice was made to ease the distinction in slight color contrast microscope images, as it might be challenging to discern the complete penetration range and evaluate it as a percentage for result comparison. Additionally, it was not possible to track the entire path of the penetration along the dentinal tubules due to their tortuous nature and the straight-cutting path. Therefore, we could not determine the complete extent of penetration around the main canal and had to rely on the maximum penetration distance after detecting all the places around the main canal.

The results show that there is a significant difference in the depth of sealer penetration when comparing SC, CLC, and WVC obturation techniques using BCHF at the coronal, middle, and apical third of the root canals of premolars, which indicated that the null hypothesis was not accepted.

The reason that the coronal, middle, and apical third among the three groups showed good DTPB is the fine particles of the BCHF (<1 µm), in addition to its basic pH which denatures the collagen fibers, its high flow rate, and its volume expansion of 0.2% after setting [14]. WVC obturation technique is characterized by the plasticization of the GP, as a result of the heat used, and after applying the heat, constant and repeated pressure is applied by the plugger that helps in condensing the GP apically and laterally, contributing to achieving a three-dimensional seal of the root canal system. This could be the reason for the maximum value of the penetration in the WVC group, which met the BCHF manufacturer’s claims that this ES becomes more flowable when exposed to heat. The superiority of the CLC obturation technique in the apical third over SC and WVC groups may be explained by two reasons. The first is that the spreader in the CLC group reaches 1 mm before the WL, which enables the practitioner to perform the lateral and apical condensation in this third effectively. This improves the pressure applied to GP and ES in the apical level, thus achieving a deeper penetration. The second reason is that the tip of the System B device does not descend more than 4-5 mm from the apex, as it was found in a study that when using the continuous wave condensation method, the highest increase in temperature was at the level of 6 mm of the root canal by 19.2°C (under normal temperature conditions 37 °C) when comparing three levels of root canal (3-6-9) mm [22]. In another study that assessed temperature levels in three sections (2-8-12) mm of the root canals, the maximum temperature was at the level of 12 mm (60°C), while at the levels of 2-8 mm, the temperature only increased by 2-6° (within the normal temperature levels of 37°C) [27].

The similarity in the DTPB values between the SC group and the WVC group at the apical third can be attributed to the fact that the SC technique relies solely on the hydraulic pressure applied by the GP cone without applying any other physical pressure either by lateral condensation or pluggers.

The coronal third achieved the highest DTPB values, regardless of the obturation technique used, which may be explained by the fact that the diameter and number of dentine tubules in the coronal third are greater than the middle third [3]. Moreover, the irrigant contact, the irrigant delivery to the coronal third, and the removal of the smear layer in the coronal third are better compared to the other two-thirds [28]. However, the middle third DTPB values were lower than the coronal third regardless of the obturation technique used, which may be explained by the fact that the diameter and number of dentin tubules in the middle third are the lowest in the premolars [3].

Finally, the apical third achieved the lowest DTPB values, regardless of the obturation technique used, which may be due to the presence of sclerotic dentin and the hardness of the smear layer in the apical third which may act as physical barriers to the penetration of the sealer [29]. It is worth noting that DTPB at the apical third was detected only under high magnifications (×2,000) with different appearances in this study.

Casino-Alegre et al. and Eid et al. compared the depth and percentage of dentinal tubule penetration with a confocal laser microscope for SC and WVC obturation techniques with BCHF in the tooth with a single canal at the coronal, middle, and apical level of the root [13,14]. They revealed that the obturation technique affect the depth and percentage of dentinal tubule penetration whereas WVC showed greater dentinal tubule penetration than the SC technique. Moreover, greater dentinal tubule penetration was observed in the coronal part compared to the apical part [13,14]. With a similar method to previous studies, Muedra et al. reported that both BS and resin-based sealers had greater dentinal tubule penetration at the coronal level using the SC technique [29]. On the other hand, Reynolds et al. followed a similar methodology to Casino-Alegre et al. and reported that a greater dentinal tubule penetration was observed at the coronal level compared to the apical level. However, the obturation technique did not affect the depth and percentage of dentinal tubule penetration [12,13].

Similarly, Dasari et al. evaluated the penetration of BioRoot RCS sealer into dentinal tubules using both CLC and WVC techniques. Their results were consistent with our findings in terms of the superiority of WVC over lateral condensation in the coronal and middle thirds only [30]. However, the results differed in the apical third between the two studies, where the WVC group showed superior penetration in the apical third. This difference could be attributed to several reasons, including the fact that Dasari et al. used a master cone matching the size of the automated taper used for preparation, which posed a challenge during condensation of the apical third in the CLC group. Additionally, cross-sectional images were evaluated using CLSM.

Limitations

This in-vitro study has some limitations, such as the challenge of achieving complete standardization, particularly regarding the number and size of dentinal tubules, where teeth with numerous and wide dentinal tubules are expected to show greater DTPB. Despite efforts to ensure balanced distribution based on the dimensions of the experimental teeth, variations in tubule characteristics remain. Moreover, the effect of the cutting method and level during the preparation of the samples may affect the image quality under SEM. Additionally, the simplicity of the single-rooted teeth used in this study did not fully represent the complexity of canal anatomy encountered in clinical cases. However, this study provides a foundational understanding and sets the stage for future research using teeth with more intricate anatomical features. Additional research should be conducted on DTPB by CLSM using Fluo-3 fluorophore and other innovative measurement methods such as penetration range instead of maximum penetration depth, as SEM did not provide sufficient color contrast to observe the complete DTPB range.

## Conclusions

Considering the limitations of this in-vitro study, the BCHF with the WVC and CLC obturation technique showed the best performance in DTPB values at the three levels. The CLC technique showed superior DTPB at the apical third compared to the WVC and SC techniques. The coronal third in each group showed higher DTPB values. Clinically, when using BCHF, it is recommended to apply either the CLC technique or the WVC technique, as they ensure a better seal of the canal system by achieving deeper penetration into the dentinal tubule.
